# Nomogram development and validation to predict Ki-67 expression of hepatocellular carcinoma derived from Gd-EOB-DTPA-enhanced MRI combined with T1 mapping

**DOI:** 10.3389/fonc.2022.954445

**Published:** 2022-10-14

**Authors:** Ziwei Liu, Shaomin Yang, Xinjie Chen, Chun Luo, Jieying Feng, Haixiong Chen, Fusheng Ouyang, Rong Zhang, Xiaohong Li, Wei Liu, Baoliang Guo, Qiugen Hu

**Affiliations:** ^1^ Department of Radiology, Shunde Hospital, Southern Medical University (The First People’s Hospital of Shunde), Foshan, China; ^2^ Department of Radiology, The Affiliated Shunde Hospital of Guangzhou Medical University, Foshan, China; ^3^ Department of Radiology, The First People’s Hospital of Foshan, Foshan, China; ^4^ Department of Radiology, The Sixth Affiliated Hospital, South China University of Technology, Foshan, China

**Keywords:** Gd-EOB-DTPA, T1 mapping, hepatocellular carcinoma, Ki-67, nomogram

## Abstract

**Objective:**

As an important biomarker to reflect tumor cell proliferation and tumor aggressiveness, Ki-67 is closely related to the high early recurrence rate and poor prognosis, and pretreatment evaluation of Ki-67 expression possibly provides a more accurate prognosis assessment and more better treatment plan. We aimed to develop a nomogram based on gadolinium ethoxybenzyl diethylenetriamine pentaacetic acid (Gd-EOB-DTPA)-enhanced magnetic resonance imaging (MRI) combined with T1 mapping to predict Ki-67 expression in hepatocellular carcinoma (HCC).

**Methods:**

This two-center study retrospectively enrolled 148 consecutive patients who underwent preoperative Gd-EOB-DTPA-enhanced MRI T1 mapping and surgically confirmed HCC from July 2019 to December 2020. The correlation between quantitative parameters from T1 mapping, ADC, and Ki-67 was explored. Three cohorts were constructed: a training cohort (n = 73) and an internal validation cohort (n = 31) from Shunde Hospital of Southern Medical University, and an external validation cohort (n = 44) from the Sixth Affiliated Hospital, South China University of Technology. The clinical variables and MRI qualitative and quantitative parameters associational with Ki-67 expression were analyzed by univariate and multivariate logistic regression analyses. A nomogram was developed based on these associated with Ki-67 expression in the training cohort and validated in the internal and external validation cohorts.

**Results:**

T1rt-Pre and T1rt-20min were strongly positively correlated with Ki-67 (r = 0.627, r = 0.607, *P* < 0.001); the apparent diffusion coefficient value was moderately negatively correlated with Ki-67 (r = -0.401, *P* < 0.001). Predictors of Ki-67 expression included in the nomogram were peritumoral enhancement, peritumoral hypointensity, T1rt-20min, and tumor margin, while arterial phase hyperenhancement (APHE) was not a significant predictor even included in the regression model. The nomograms achieved good concordance indices in predicting Ki-67 expression in the training and two validation cohorts (0.919, 0.925, 0.850), respectively.

**Conclusions:**

T1rt-Pre and T1rt-20min had a strong positive correlation with the Ki-67 expression in HCC, and Gd-EOB-DTPA enhanced MRI combined with T1 mapping-based nomogram effectively predicts high Ki-67 expression in HCC.

## Introduction

Hepatocellular carcinoma (HCC), as the fifth most common malignancy and the fourth leading cause of cancer-related deaths worldwide, is the most common primary malignant tumor of the liver with increasing incidence (1, 2). The main treatments for HCC include surgical resection, liver transplantation, and transarterial chemoembolization, among others. Surgical resection is recognized as an early radical treatment method, but the recurrence rate of 5 years after surgical resection is as high as 60%–70% (3–5). High early recurrence rate is an important factor affecting the long-term survival and poor prognosis of patients with HCC. The immunohistochemical marker Ki-67 is a nuclear antigen related to cell proliferation activity, and it is a common indicator that reflects the level of cell proliferation. Ki-67 has been proposed as the most valuable independent predictor for evaluating early recurrence and poor prognosis of surgically resected HCC in recent studies (6–8). Ki-67 detection relies on pathological examination. Needle biopsy is a common method to obtain pathological tissue, but it is an invasive examination and has certain disadvantages, such as poor patient compliance, possible surgical risks, and needle tract transfer (9, 10). With the formation of a multidisciplinary and multimethod comprehensive treatment model for liver cancer, a non-invasive preoperative method to predict Ki-67 status is significant in the treatment and prognostic management of patients.

Gadolinium-ethoxybenzyl-diethylenetriamine pentaacetic acid (Gd-EOB-DTPA) is a liver-specific contrast agent, which has dual properties extracellular and hepatobiliary that can be taken up by normal liver cells and is increasingly used in the diagnosis and evaluation of liver diseases. T1 mapping is a non-invasive method of quantitatively analyzing the T1 value of tissue reflecting the T1 relaxation time. T1 mapping combined with GD-EOB-DTPA can provide more accurate and objective magnetic resonance imaging (MRI) quantitative images with functional information. Previous studies have shown that T1 mapping can effectively predict the postoperative histopathological grade and recurrence status of HCC (11, 12) and effectively assess liver fibrosis (13) and liver function (14, 15). These provide a basis for the non-invasive preoperative prediction of Ki-67 expression. To our knowledge, few studies had reported the application of T1 mapping combined with GD-EOB-DTPA for the evaluation of Ki-67 expression in HCC.

Therefore, the purpose of this study was to investigate the correlation between quantitative parameters from T1 mapping, apparent diffusion coefficient (ADC), and Ki-67 expression. We aimed to develop a nomogram based on Gd-EOB-DTPA-enhanced MRI combined with T1 mapping to non-invasive preoperatively predict Ki-67 expression in HCC.

## Materials and methods

### Patients

**Figure 1:**
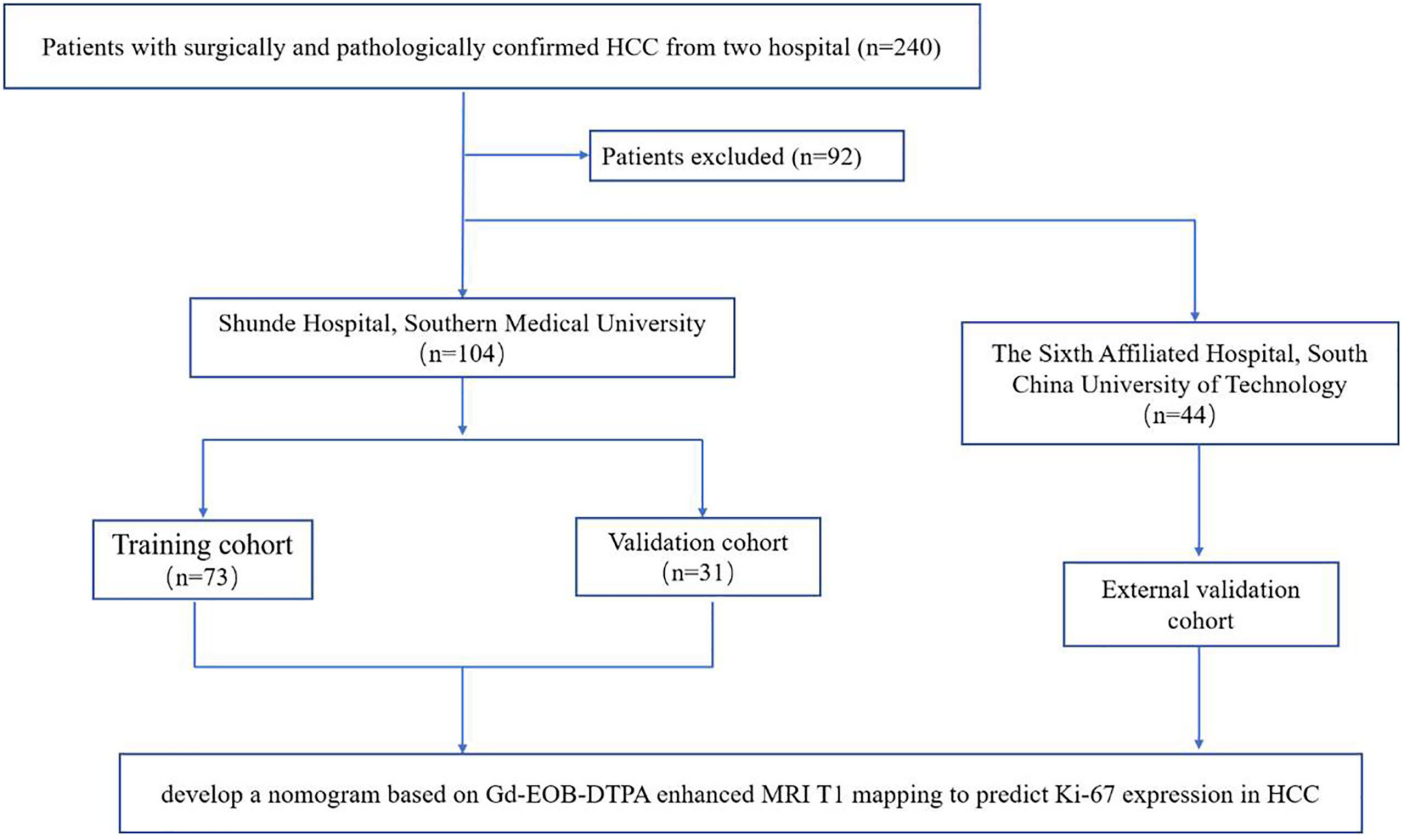
Flowchart of patient selection in the study.

This two-center retrospective study was approved by the ethics committee of each participating hospital and was conducted in accordance with the Declaration of Helsinki. The informed consent had a waiver of informed consent. The entire workflow of this retrospective study is shown in **Figure 1**. A total of 240 patients with surgically and pathologically confirmed HCC from two hospital centers (Shunde Hospital of Southern Medical University, and the Sixth Affiliated Hospital, South China University of Technology) were collected between July 2019 and December 2020. Finally, 148 patients with preoperative Gd-EOB-DTPA-enhanced MRI T1 mapping and clinical data were included in the final analysis, according to the inclusion and exclusion criteria. Three cohorts were constructed: a training cohort (n = 73) and an internal validation cohort (n = 31) from Shunde Hospital of Southern Medical University, and an external validation cohort (n = 44) from the Sixth Affiliated Hospital, South China University of Technology. The inclusion criteria were as follows: (a) pathologically confirmed solitary HCC; (b) underwent hepatectomy; (c) and received preoperative Gd-EOB-DTPA-enhanced MRI and the interval period between the MRI examination and operation in less than 2 weeks. The exclusion criteria were as follows: (a) received previous treatment; (b) incomplete clinical or pathological information; (c) incomplete T1 mapping image data (pre-enhanced and 20 min after enhancement); (d) incomplete MR images or poor quality with obvious artifact; (e) and multiple HCC (≥2).

One hundred four patients in the internal cohort were followed up. We followed up patients after surgical resection every 3–6 months by contrast-enhanced ultrasound, CT, or MRI. We defined early recurrence (ER) as recurrence within 1 year after surgery.

### Histopathological examination

All tissue specimens were sectioned and stained with hematoxylin eosin, and Ki-67 immunohistochemical staining was performed in the pathology department from two hospitals, respectively. The histopathological and immunohistochemical marker Ki-67 was blinded performed by two experienced pathologists from two hospitals (10, 12, 14, and 16 years’ experience, respectively). In case of disagreements, a third senior pathologist was consulted.

**Figure 2:**
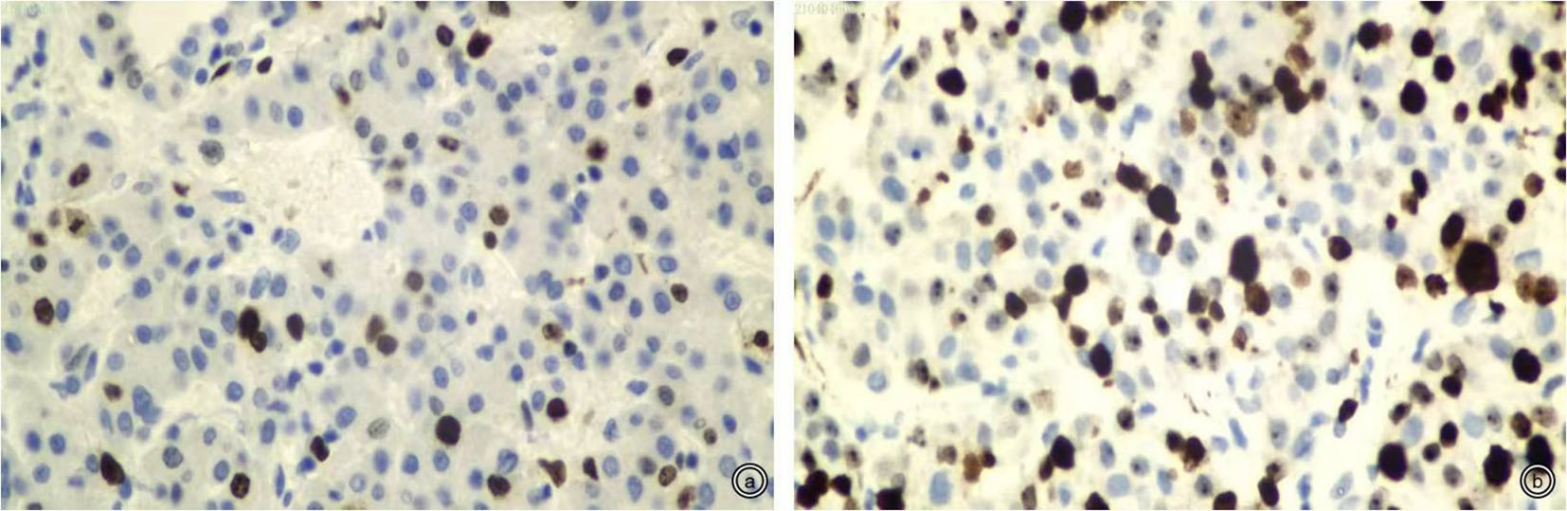
Representative staining patterns of Ki-67 (×400). **(A, B)**: Low and high Ki-67 expression (approximately 10% and 50%).

Histological differentiation was assessed by using the Edmondson–Steiner grading system. If different tumor grades coexist within a tumor, the diagnosis is made with the highest grade. Moreover, Edmondson–Steiner grades were divided into two groups; grades 1 and 2 were defined as high differentiation, and grades 3 and 4 were defined as low differentiation (16). Immunohistochemical staining was checked for Ki-67 expression (Beijing Zhongshan Golden Bridge Biotechnology Company, Beijing, China). Interpretation of Ki-67 by immunohistochemistry was as follows: take five FOVs under a high-power microscope (×400), count 100 cells for each FOV, and count the positively stained cells. Ki-67 was identified as the percentage of positive cells to the total cells, and the average value was used and was classified as low Ki-67 expression (≤25%) or high Ki-67 expression (>25%) according to previous studies (6, 17–19) (**Figure 2**).

### MRI examination

All MRI examinations from two hospitals were performed on a 3.0-T MR scanner (MAGNETOM Skyra or MAGNETOM Verio, Siemens Healthcare, Germany). All patients should be fasted for ≥6 h before the examination, and the patients should be trained in breathing before the scan. The examination range was from the upper edge of the liver to the lower edge of the liver. The main scanning sequence includes T1WI-volumetric interpolated breath-hold examination (VIBE), T1WI-TWIST-VIBE sequence, T2WI-BLADE, T2WI-HASTE (half Fourier single-shot turbo spin echo), diffusion-weighted imaging (DWI), and T1 mapping. The parameters are detailed in **Table 1**.

**Table 1:** MRI scan sequence and parameters.

Hospital	Scanner	Sequence	TR(ms)	TE(ms)	Fov(mm)	Matrix	Reverse angle	Seam thickness (mm)	Fat suppression mode
Shunde Hospital, Southern Medical University	Skyra	T1WI-VIBE	4	1.3/2.5	380×380	240×320	9°	3	Dixon
		T1WI-TWIST-VIBE	3.89	1.2/2.5	400×320	216×288	10°	3	Dixon
		T2WI-BLADE	2,000	84	380×380	320×320	90°	5	Spair
		DWI	6,200	50	285×380	128×128	/	5	Spair
		T1 mapping	5.01	2.3	285×380	168×224	3°/15°	4	/
The Sixth Affiliated Hospital, South China University of Technology	Verio	T1WI-VIBE	3.4	1.3/2.6	328×350	228×256	13°	3	Dixon
		T2WI-HASE	1,300	97	360×280	256×320	160°	6	/
		DWI	5,100	73	360×288	154×192	/	5	Fat sat
		T1 mapping	4.2	1.4	273×380	161×320	5°/15°	3	/

VIBE, volumetric interpolated breath-hold examination; DWI, diffusion-weighted imaging; HASE, half Fourier single-shot turbo spin echo.

GD-EOB-DTPA (Primovist, Bayer Schering Pharma, Berlin, German) was used for enhanced scanning, the dose was 0.1 ml/kg, the flow rate was 1.0 ml/s, and the tube was flushed with 30 ml of physiological saline. The T1WI-VIBE or T1WI-TWIST-VIBE sequence was used for multiphase (two to five phases) arterial phase scanning 10–30 s after spraying, and the T1WI-VIBE sequence was used for portal vein and balance phase scanning 60 and 150 s after spraying. Hepatobiliary imaging after enhancement was performed 20 min later. T1 mapping pre-enhancement was performed before enhancement, and T1 mapping after enhancement was performed 20 min later.

### Imaging analysis

The patients’ image data were exported from the PACS in the DICOM format, and the RadiAnt DICOM Viewer 2020.2 (https://www.radiantviewer.com) software was used for image reading. All MRI quantitative and qualitative features were independently assessed by two abdominal radiologists from Shunde Hospital of Southern Medical University (8 and 15 years’ experience, respectively) who were blinded to the patients’ clinical and pathological information. If there is disagreement on the reevaluated image, it was resolved by consensus.

**Figure 3:**
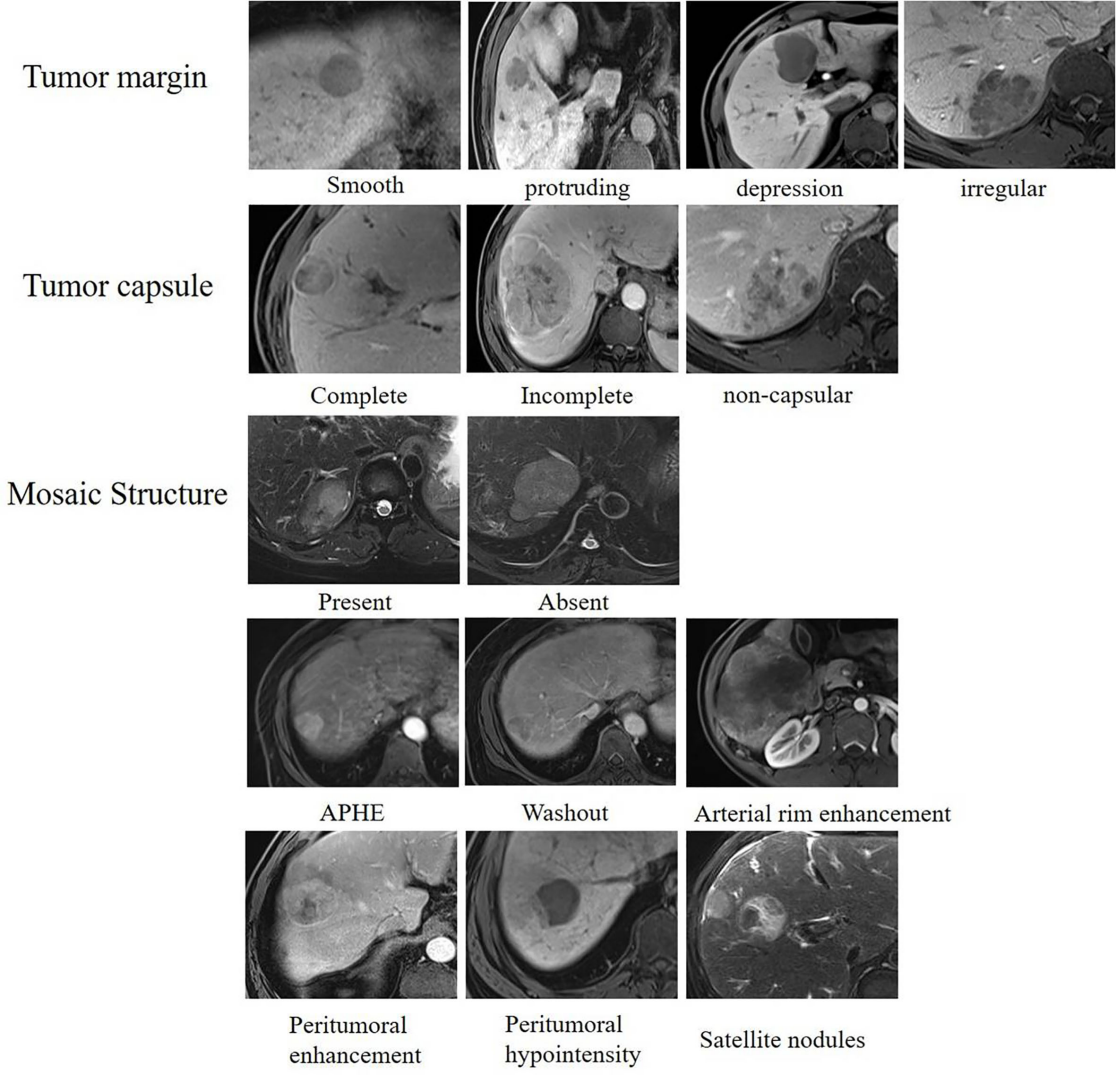
Qualitative features of hepatocellular carcinoma. Assessment of qualitative features including tumor margin, tumor capsule, mosaic structure, APHE, washout, arterial rim enhancement, peritumoral enhancement, peritumoral hypointensity, and satellite nodules.

The following qualitative features were evaluated (**Figure 3**): (1) tumor margin, including smooth (round or oval with smooth margin) and non-smooth margins (protrusion, depression, or irregular); (2) tumor capsule, defined as the peripheral rim of hyperintensity in the equilibrium phase and classified as complete capsule, and incomplete or non-capsular; (3) mosaic structure, defined as the presence of randomly distributed internal nodules or compartments in the T2-weighted images; (4) arterial phase hyperenhancement (APHE) was defined as enhancement in the arterial phase unequivocally greater in part or in whole than the liver (no non-rim APHE excluded); (5) arterial rim enhancement, defined as the arterial phase enhancement which is most pronounced in the observation periphery; (6) peritumoral enhancement, defined as peri-observation enhancement in the late arterial phase or early portal phase; (7) peritumoral hypointensity, defined as the wedge-shaped area or irregular around the tumor with hypointensity in the HBP (slightly higher signal intensity than the tumor and lower than the surrounding normal liver parenchyma); and (8) satellite nodules, defined as tumors ≤2 cm in size and located ≤2 cm from the main tumor.

**Figure 4:**
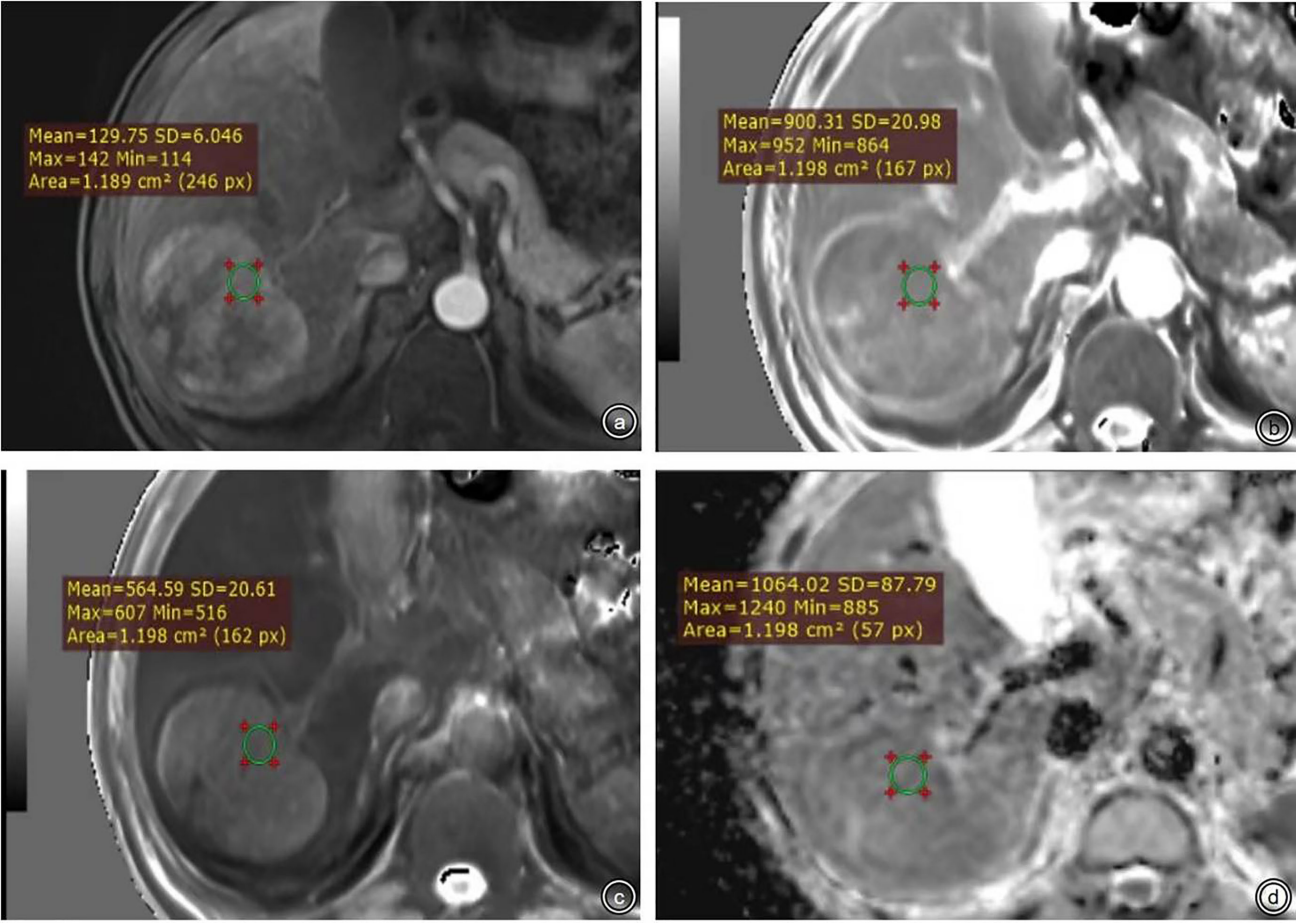
**(A-D)** were ROI measurement methods of T1 relaxation time obtained from pre-enhanced T1 mapping and 20 min after enhancement and the ADC map.

The quantitative characteristics included tumor size, T1 relaxation time obtained from T1 mapping pre-enhancement and 20 min after enhancement (T1rt-Pre, T1rt-20min), and ADC value. Selection of the region of interest (ROI) was conducted as follows: (1) select the largest level of measurement as much as possible, and keep the same level of each sequence as much as possible and ROI should be placed as far as possible in the area of obvious enhancement of the lesion; if the tumor is larger than 5 cm, the method of averaging multiple ROIs is used (avoid hemorrhage, cystic area, and blood vessels; **Figure 4**). (2) The ROIs were not smaller than 1.0 cm^2^ (100–150 pixels); the same lesion was measured three times with the same ROI, and then average amounts were calculated. The average values of the measurements of two radiologists were used for further analyses. (3) The following data were measured: T1 relaxation time of pre-enhancement (T1rt-Pre) and T1 relaxation time 20 min after enhancement (T1rt-20min) on the T1 mapping; calculate the reduction rate of T1 relaxation time (rrT1rt): rrT1rt-20min = (T1rt-Pre-T1rt-20min)/T1rt-Pre; ADC value on the ADC map; and tumor diameter on the maximum diameter of the tumor measured in the transverse or coronal view.

### Statistical analysis

Quantitative variables were expressed as mean and standard deviation (SD) (if normally distributed) and median and range (if non-normally distributed), while categorical data were presented as counts and proportions. The independent-sample t-test or Mann–Whitney U-test were used to assess the continuous variables, while the chi-squared test or Fisher’s exact test was used to analyze the categorical variables. Test–retest reliability was assessed using the intraclass correlation coefficient (ICC) for quantitative measurements and kappa for MRI features (>0.75 was considered to represent good agreement). The Spearman correlation analysis method was used to analyze the relationship between quantitative parameters from T1 mapping and ADC and Ki-67 expression in the whole cohort.

Patients from the internal cohort were randomly divided into the training and validation cohorts at a ratio of 7:3. The MRI features and clinical data were compared respectively between the training *vs*. validation cohorts in the internal cohort and between the Ki-67 high expression group and the low expression group in the internal cohort and the external validation cohort. The variables with statistical differences were determined by univariate logistic regression analysis. Then, the above-identified variables were further selected by stepwise regression based on the Akaike information criterion to construct a multivariate logistic regression model for Ki-67 expression. The nomogram was constructed based on the corresponding final logistic model prediction for these features and was separately validated with the same validation dataset and external dataset. We used Harrell’s C-index and calibration curves in the training and validation cohorts to test the performance of the model. We also calculated the area under the characteristic operating curves and 95% confidence intervals (CIs), accuracy, sensitivity, specificity, positive predictive value, and negative predictive value. Kaplan–Meier analysis was used to compare early recurrence between high Ki-67 expression groups and low Ki-67 expression groups. Statistical significance was considered as a two-sided p value of less than 0.05. The above statistical analysis was performed by the R software version 3.2.3 (Bell Laboratories; https://cran.r-project.org/bin/windows/base/old/3.2.3).

## Results

### Correlation analysis of quantitative parameters from T1 mapping, ADC and Ki-67 expression, and histological differentiation

**Figure 5:**
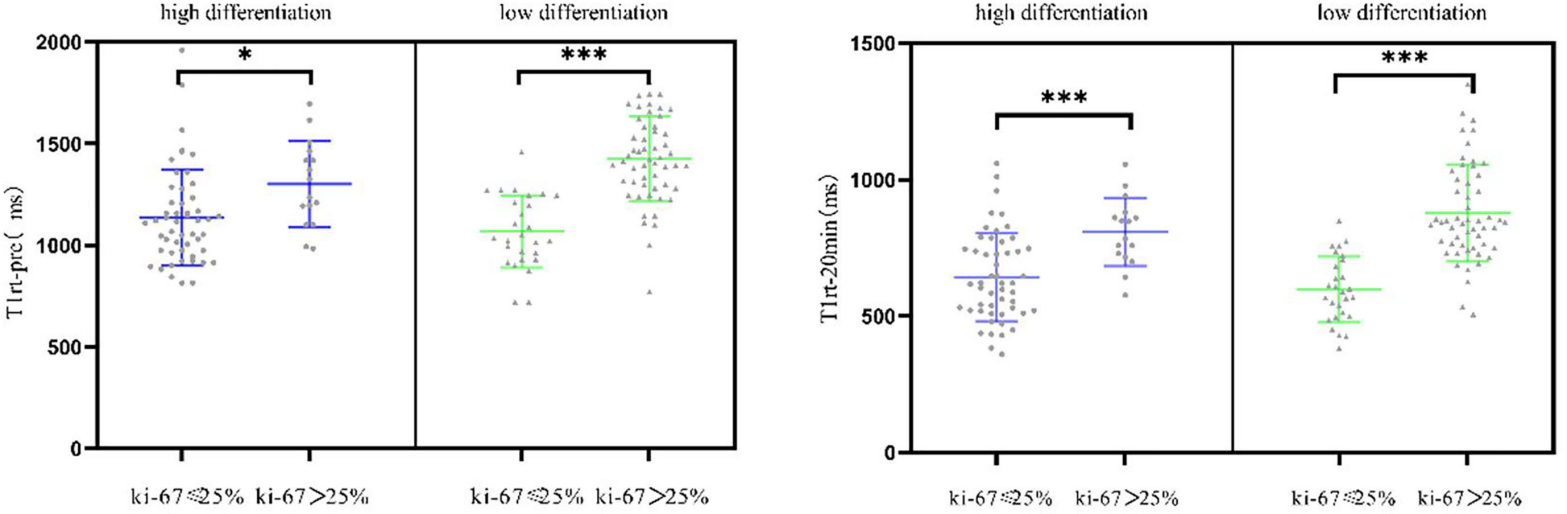
Correlation between the quantitative parameters of T1 mapping with Ki-67 expression in different histological differentiation patients (*<0.05; ***<0.001).

T1rt-Pre and T1rt-20min are strongly positively correlated with Ki-67 (r = 0.627, r = 0.607, *P* < 0.001); the ADC value was moderately negatively correlated with Ki-67 (r = -0.401, *P* < 0.001). rrT1rt-20min was not significantly associated with Ki-67 (r = -0.150, *P* = 0.069). The correlation between T1rt-Pre and T1rt-20min with Ki-67 expression in different histological differentiation patients are shown in **Figure 5** (all *P* < 0.05).

### Baseline characteristics

**Figure 6:**
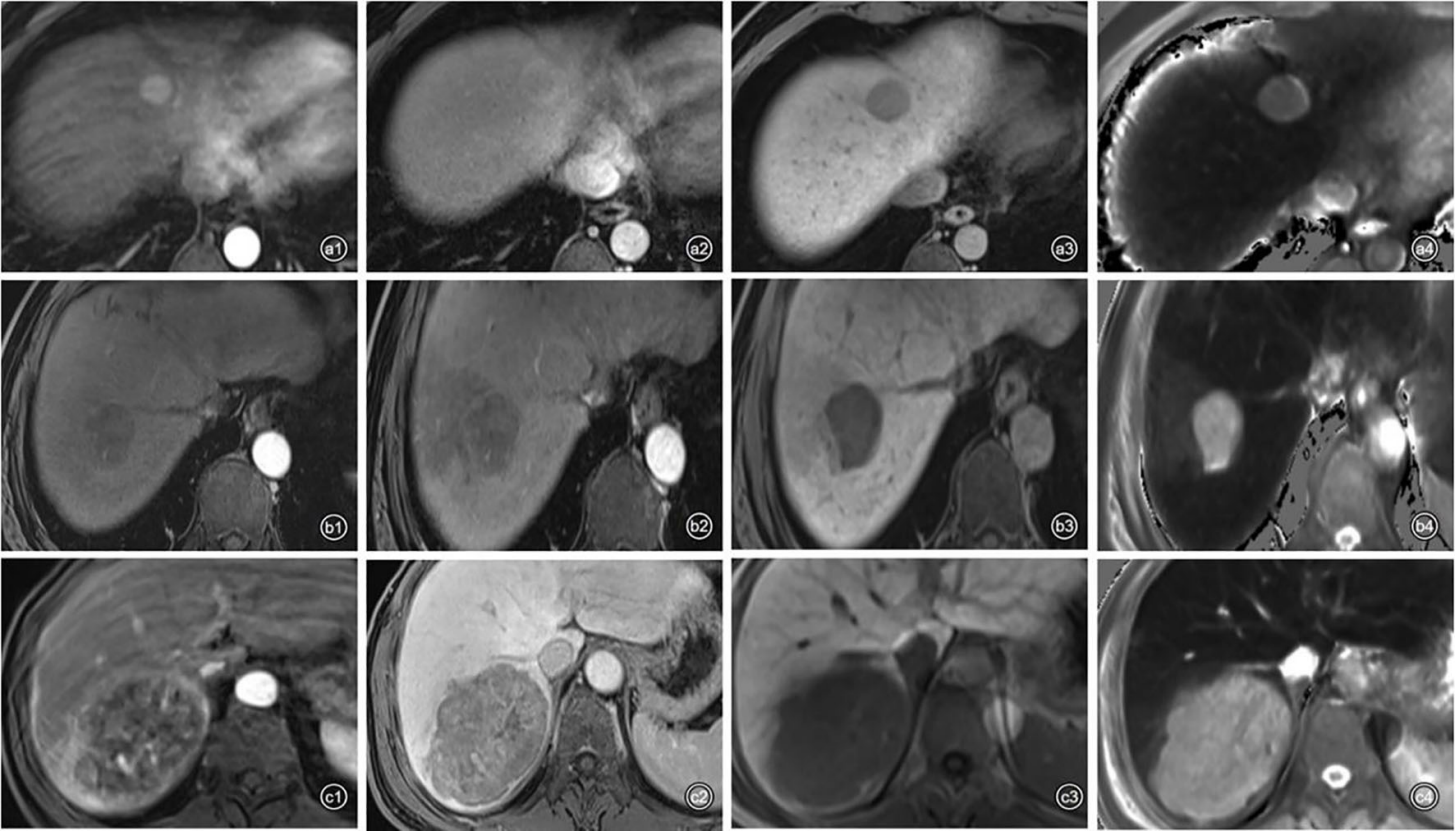
Representative images of each phase on Gd-EOB-DTPA-enhanced MRI and T1 mapping 20 min after enhancement in patients with high or low Ki-67 expression. a1–4: A 48-year-old man with Edmondson–Steiner grade II HCC and low expression of Ki-67 (10%). (a1) APHE; (a2), washout and complete capsule; (a3), smooth tumor margin; (a4), T1rt-20 min = 509.92 ms. b1–4: A 58-year-old man with Edmondson–Steiner grade III HCC and high expression of Ki-67 (30%). (b1) no APHE; (b2), peritumoral enhancement; (b3), peritumoral hypointensity and non-smooth tumor margin; (b4), T1rt-20 min = 1,062.86 ms. c1–4: A 59-year-old man with Edmondson–Steiner grade II HCC and high expression of Ki-67 (40%). (c1), rim enhancement and peritumoral enhancement; (c2), washout; (c3), non-smooth tumor margin; (c4), T1rt-20min = 971.84 ms.

From July 2019 to December 2020, 104 consecutive patients were initially enrolled from the internal cohort, with 73 patients assigned to the training cohort and 31 to the validation cohort (**Table 2**). There was no significant difference between the two cohorts in any of the listed variables in **Table 2** (all P > 0.05). Among the internal cohort, 50 patients had a high Ki-67 expression (48.1%) and 54 showed a low Ki-67 expression (51.9%). Regarding quantitative data, patients with a high Ki-67 expression had higher alpha-fetoprotein (AFP) levels, larger tumor size, higher T1rt-pre, higher T1rt-20min, and lower ADC (all *P* < 0.05, **Table 3**). Regarding qualitative data, patients with high Ki-67 expression more frequently showed APHE, a non-smooth margin, an incomplete or non-complete tumor capsule, a mosaic structure, arterial rim enhancement, peritumoral enhancement, and peritumoral hypointensity (all *P* < 0.05, **Table 3**). Among the external validation cohort, 20 patients had a high Ki-67 expression (45.5%) and 24 showed a low Ki-67 expression (54.5%). Patients with a high Ki-67 expression had higher albumin levels, larger tumor size, higher T1rt-pre, higher T1rt-20min, and lower ADC (all *P* < 0.05, **Table 3**). Regarding qualitative data, patients with high Ki-67 expression more frequently showed a non-smooth margin, mosaic structure, arterial rim enhancement, peritumoral enhancement, and peritumoral hypointensity (all *P* < 0.05, **Table 3**). There were no statistically significant differences in other MR features and clinical indicators between the two groups (*P* > 0.05). The average concordance rate of evaluating MRI features between the two radiologists was 0.877 (95% CI, 0.855–0.899). MRI images of typical cases in the high and low Ki-67 expression groups are shown in **Figure 6**.

**Table 2:** Baseline characteristics between patients in the internal training and validation cohorts.

Variables	Total (n = 104)	Training cohort (n = 73)	Validation cohort (n = 31)	*P* value
Sex				1.000
Male	91 (87.5%)	64 (87.7%)	27 (87.1%)	
Female	13 (12.5%)	9 (12.3%)	4 (12.9%)	
Age (years)				0.543
≤55	39 (37.5%)	21 (28.8%)	18 (58.1%)	
>55	65 (62.5%)	52 (71.2%)	13 (41.9%)	
Hepatitis				0.382
Absent	16 (15.4%)	13 (17.8%)	3 (9.7%)	
Present	88 (84.6%)	60 (82.2%)	28 (90.3%)	
AFP (ng/mL)				0.733
≤20	63 (60.6%)	45 (61.6%)	18 (58.1%)	
>20	41 (39.4%)	28 (38.4%)	13 (41.9%)	
ALT (U/L)	37.50 (23.00, 64.75)	38.00 (23.05, 65.00)	34.00 (18.00, 65.00)	0.520
AST (U/L)	36.00 (26.00, 53.75)	36.00 (26.00, 55.50)	35.00 (22.00, 50.10)	0.534
GGT (U/L)	52.00 (35.25, 99.75)	56.00 (35.00, 109.05)	46.00 (36.00, 64.00)	0.120
PLR	117.70 (78.80, 150.65)	114.29 (72.89, 150.65)	125.49 (90.32, 171.01)	0.117
NLR	2.21 (1.58, 3.14)	2.17 (1.57, 2.90)	2.62 (1.60, 3.79)	0.354
Albumin (g/L)	42.35 (38.30, 44.65)	42.50 (38.65, 44.60)	41.00 (37.60, 44.90)	0.717
Child–Pugh				0.522
A	91 (87.5%)	65 (89.0%)	26 (83.9%)	
B	13 (12.5%)	8 (11.0%)	5 (16.1%)	
APHE				0.547
Absent	14 (13.5%)	11 (15.1%)	3 (9.7%)	
Present	90 (86.5%)	62 (84.9%)	28 (90.3%)	
Washout				0.721
Absent	9 (8.7%)	7 (9.6%)	2 (6.5%)	
Present	95 (91.3%)	66 (90.4%)	29 (93.5)	
Tumor margin				0.834
Smooth	42 (40.4%)	29 (39.7%)	13 (41.9%)	
Non-smooth	62 (59.6%)	44 (60.3%)	18 (58.1%)	
Tumor capsule				0.131
Complete	45 (43.3%)	27 (37.0%)	18 (58.1%)	
Incom-/non	59 (56.7%)	46 (63.0%)	13 (41.9%)	
Mosaic structure				0.759
Absent	46 (44.2%)	33 (45.2%)	13 (41.9%)	
Present	58 (55.8%)	40 (54.8%)	18 (55.8%)	
Arterial rim enhancement				0.821
Absent	79 (76.0%)	55 (75.3%)	24 (77.4%)	
Present	25 (24.0%)	18 (24.7%)	7 (22.6%)	
Peritumoral enhancement				0.733
Absent	63 (60.6%)	45 (61.6%)	18 (58.1%)	
Present	41 (39.4%)	28 (38.4%)	13 (41.9%)	
Peritumoral hypointensity				0.398
Absent	71 (68.3%)	48 (65.8%)	23 (74.2%)	
Present	33 (31.7%)	25 (34.2%)	8 (25.8%)	
Satellite nodules				0.437
Absent	79 (76.0%)	57 (78.1%)	22 (71.0%)	
Present	25 (24.0%)	16 (21.9%)	9 (29.0%)	
Tumor diameter (cm)	3.80 (2.33, 6.05)	3.60 (2.20, 6.30)	3.90 (2.60, 5.90)	0.619
T1rt-pre (ms)	1,232.20 (1014.64, 1450.03)	1,225.63 (1003.68, 1441.25)	1,271.36 (1014.64, 1462.63)	0.402
T1rt-20min (ms)	748.92 ± 201.00	745.65 ± 191.38	755.91 ± 225.22	0.819
rrT1rt-20min	0.396 ± 0.124	0.389 ± 0.117	0.412 ± 0.140	0.384
ADC (mm^2^/s)	1,081.86 ± 185.43	1081.23 ± 184.35	1083.33 ± 191.04	0.985
Histological differentiation				0.574
High	48 (46.2%)	35 (47.9%)	13 (41.9%)	
Low	56 (53.8%)	38 (52.1%)	18 (58.1%)	
Ki-67 expression	25.00 (10.00, 40.00)	25.00 (10.00, 40.00)	25.00 (10.00, 45.00)	0.391
Ki-67 group				0.967
≤25%	54 (51.9%)	38 (52.1%)	16 (51.6%)	
>25%	50 (48.1%)	35 (47.95)	15 (48.4%)	

Continuous variables with normal distribution are presented as mean (standard deviation, SD), and those with abnormal distribution as median (interquartile range, IQR). Categorical variables are presented as N (%) according to different levels. (AFP, alpha-fetoprotein; ALT, alanine aminotransferase; AST, aspartate aminotransferase; GGT, galactosyl glucosyltransferase; NLR, neutrophil-to-lymphocyte ratio; PLR, platelet-to-lymphocyte ratio; APHE, arterial phase hyperenhancement; ADC, apparent diffusion coefficient).

**Table 3:** Baseline characteristics of patients in the Ki-67 high and low expression groups.

	Internal cohort (n = 104)				External validation cohort (n = 44)			
Variables	Kappa/ICC	High Ki-67 group (n = 50)	Low Ki-67 group (n = 54)	*P* value	Kappa/ICC	High Ki-67 group (n = 20)	Low Ki-67 group (n = 24)	*P* value
Sex	/			0.182	/			0.493
Male		46 (92.0%)	45 (83.3%)			20 (100.0%)	22 (91.7%)	
Female		4 (8.0%)	9 (16.7%)			0 (0.0%)	2 (8.3%)	
Age (years)	/			0.188	/			0.956
≤55		22 (44.0%)	17 (31.5%)			9 (45.0%)	11 (45.8%)	
>55		28 (56.0%)	37 (68.5%)			11 (55.0%)	13 (54.2%)	
Hepatitis	/			0.143	/			0.614
Absent		5 (10.0%)	11 (20.4%)			1 (5.0%)	3 (12.5%)	
Present		45 (90.0%)	43 (79.6%)			19 (95.0%)	21 (87.5%)	
AFP (ng/mL)	/			<0.001	/			0.069
≤20		19 (38.0%)	44 (81.5%)			7 (35.0%)	15 (62.5%)	
>20		31 (62.0%)	10 (18.5%)			13 (65.5%)	9 (37.5%)	
ALT (U/L)	/	39.50 (24.75, 67.00)	32.50 (19.60, 57.50)	0.127	/	34.00 (22.50,68.25)	25.00 (14.25, 43.50)	0.150
AST (U/L)	/	36.00 (27.75, 54.00)	34.50 (23.75, 51.50)	0.276	/	38.50 (26.25,46.00)	39.00 (31.00,49.50)	0.595
GGT (U/L)	/	52.00 (42.00, 90.05)	53.00 (34.75, 101.00)	0.800	/	49.50 (43.25,129.25)	60.00 (33.25, 100.00)	0.724
PLR	/	128.34 (90.17, 150.65)	109.28 (72.83, 152.72)	0.112	/	98.60 (68.57,141.34)	100.86 (59.10, 132.53)	0.671
NLR	/	2.43 (1.59, 3.71)	2.10 (1.54, 2.87)	0.373	/	1.68 (1.15,2.70)	2.15 (1.43, 2.58)	0.289
Albumin (g/L)	/	42.00 (39.00, 43.80)	42.75 (37.80,45.7)	0.524	/	41.45 (38.73,46.63)	37.85 (32.65, 42.65)	0.011
Child–Pugh	/			0.458	/			0.246
A		45 (90.0%)	46 (85.2%)			15 (75.0%)	14 (58.3%)	
B		5 (10.0%)	8 (14.8%)			5 (25.0%)	10 (41.7%)	
APHE	0.922			0.007	0.927			0.117
Absent		2 (4.0%)	12 (22.2%)			2 (10.0%)	7 (29.2%)	
Present		48 (96.0%)	42 (77.8%)			18 (90.0%)	17 (70.8%)	
Washout	0.889			0.819	0.864			0.545
Absent		4 (8.0%)	5 (9.3%)			9 (45.0%)	13 (54.2%)	
Present		46 (92.0%)	49 (90.7%)			11 (55.0%)	11 (45.8%)	
Tumor margin	0.939			<0.001	0.909			<0.001
Smooth		6 (12.0%)	36 (66.7%)			3 (15.0%)	19 (79.2%)	
Non-smooth		44 (88.0%)	18 (33.3%)			17 (85.0%)	5 (20.8%)	
Tumor capsule	0.806			0.027	0.800			0.469
Complete		17 (34.0%)	30 (55.6%)			13 (65.0%)	18 (75.0%)	
Incom-/non		33 (66.0%)	24 (44.4%)			7 (35.0%)	6 (25.0%)	
Mosaic structure	0.942			<0.001	0.909			0.008
Absent		13 (26.0%)	33 (61.1%)			7 (35.0%)	18 (75.0%)	
Present		37 (74.0%)	21 (38.9%)			13 (65.0%)	6 (25.0%)	
Arterial rim enhancement	0.924			<0.001	0.899			0.018
Absent		30 (60.0%)	49 (90.7%)			10 (50.0%)	20 (83.3%)	
Present		20 (40.0%)	5 (9.4%)			10 (50.0%)	4 (16.7%)	
Peritumoral enhancement	0.816			<0.001	0.782			0.016
Absent		18 (36.0%)	45 (83.3%)			11 (55.0%)	21 (87.5%)	
Present		32 (64.0%)	9 (16.7%)			9 (45.0%)	3 (12.5%)	
Peritumoral hypointensity	0.802			<0.001	0.771			0.001
Absent		25 (50.0%)	46 (85.2%)			10 (50.0%)	23 (95.8%)	
resent		25 (50.0%)	8 (14.8%)			10 (50.0%)	1 (4.2%)	
Satellite nodules	0.922			0.068	0.871			0.057
Absent		34 (68.0%)	45 (83.3%)			13 (65.0%)	22 (91.7%)	
Present		16 (32.0%)	9 (16.7%)			7 (35.0%)	2 (8.3%)	
Tumor diameter (cm)	0.986	4.80 (3.32, 7.43)	2.65 (1.60, 4.25)	<0.001	0.950	40.10 (26.28,59.03)	30.95 (19.30, 39.18)	0.044
T1rt-pre (ms)	0.862	1,425.24 (,1244.69, 1,592.75)	1,042.40 (931.58, 1,215.96)	<0.001	0.821	1360.00 (1250.55,1463.00)	1,132.40 (1,037.68, 1,279.00)	0.004
T1rt-20min (ms)	0.872	876.67 ± 157.62	630.63 ± 160.91	<0.001	0.839	825.39 ± 191.03	620.53 ± 121.77	<0.001
rrT1rt-20min	0.920	0.377 ± 0.106	0.414 ± 0.137	0.125	0.894	0.39 ± 0.12	0.46 ± 0.12	0.053
ADC (mm^2^/s)	0.839	1,008.02 ± 147.06	1,150.22 ± 192.16	<0.001	0.880	923.30 (844.28,1013.50)	1,081.50 (979.25, 1,286.35)	0.008

Continuous variables with normal distribution are presented as mean (standard deviation, SD), and those with abnormal distribution as median (interquartile range, IQR). Categorical variables are presented as N (%) according to different levels. (AFP, alpha-fetoprotein; ALT, alanine aminotransferase; AST, aspartate aminotransferase; GGT, galactosyl glucosyltransferase; NLR, neutrophil-to-lymphocyte ratio; PLR, platelet-to-lymphocyte ratio; APHE, arterial phase hyperenhancement; ADC, apparent diffusion coefficient).

**Figure 7:**
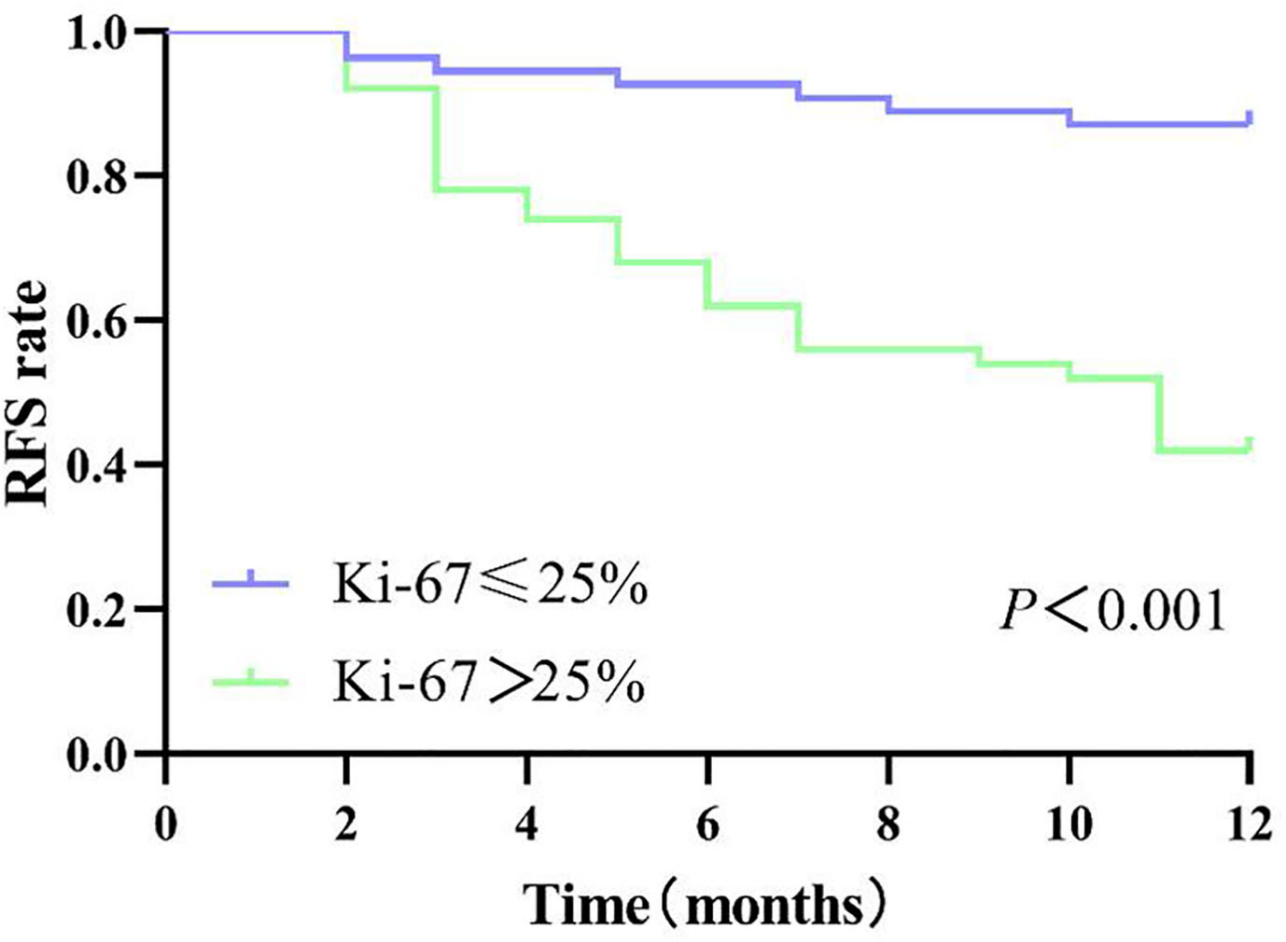
Disease-free survival of patients with high Ki-67 expression and low Ki-67 expression.

Patients in the internal cohort were followed up until recurrence or at the end point of this study (1 February 2022). The median follow-up time of the patients was 18.96 (range 17.07–20.86) months. The overall early recurrence rate was 34.62% (36/104) and in particular 23.32 (21.44–25.19) months for those with low Ki-67 and 13.84 (11.13–16.56) months for those with high Ki-67 (log-rank test, *P*<0.001; **Figure 7**).

### Performance of predictors and the combined nomogram for predicting high Ki-67 expression

Using multivariable analyses, we found significant independent predictors of Ki-67 expression, including peritumoral enhancement, peritumoral hypointensity, T1rt-20min, and tumor margin, while APHE was marginally significant predictors of Ki-67 expression (**Table 4**).

**Table 4:** Multivariable analyses of preoperative Gd-EOB-DTPA enhanced MRI features in prediction of Ki-67 expression.

Variables	OR	95% CI	*P* value
APHE	17.77	0.48∼663.93	0.119
Peritumoral enhancement	7.97	1.24∼51.38	0.029
Peritumoral hypointensity	0.09	0.01∼0.86	0.037
T1rt-20min (ms)	1.01	1.00∼1.02	0.001
Tumor margin	9.52	1.76∼51.39	0.009

APHE, arterial phase hyperenhancement.

**Figure 8:**
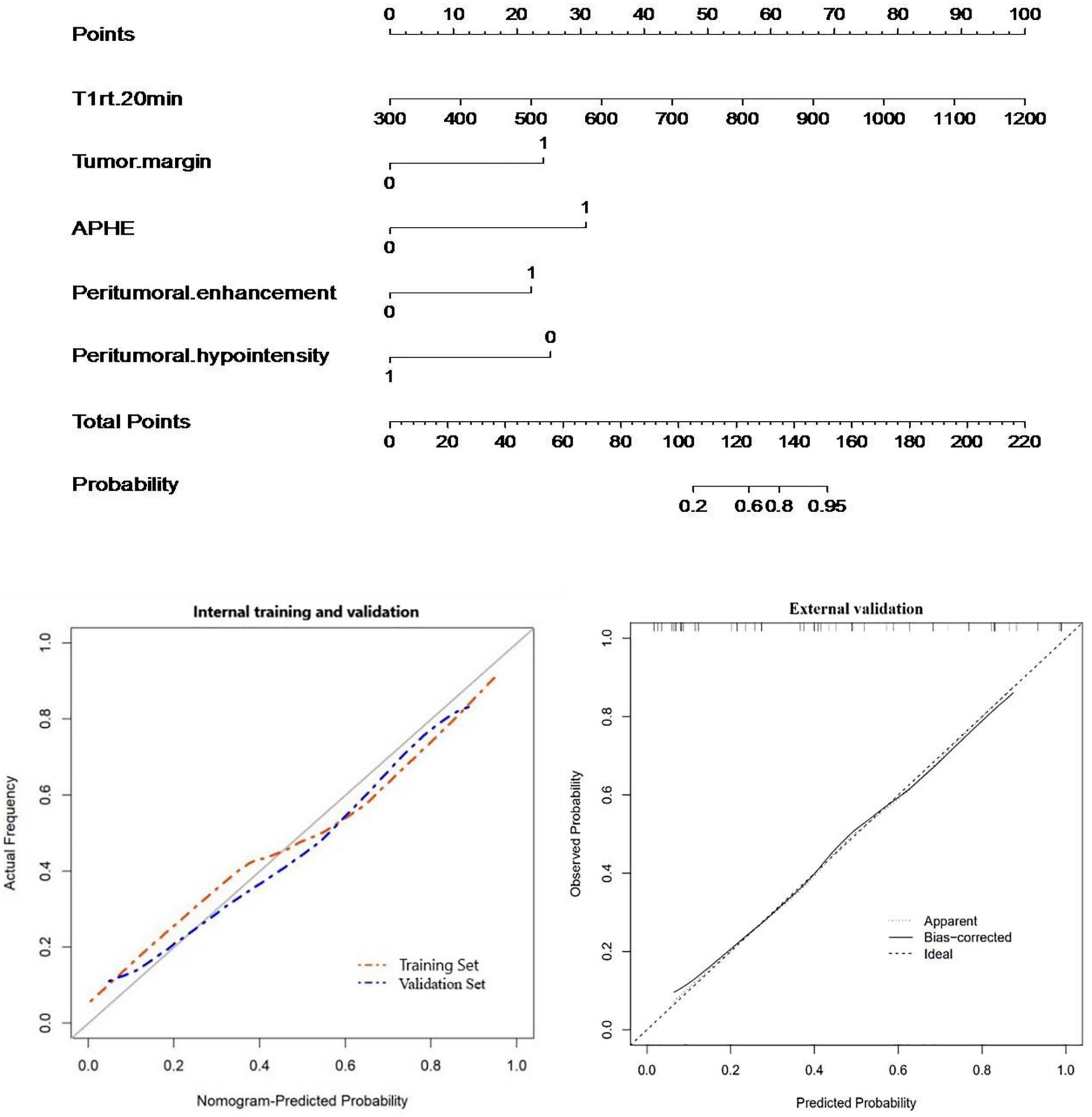
Nomogram with calibration curves for predicting Ki-67 expression (internal cohort and external validation cohort).

For Ki-67 expression, a nomogram was established in the training cohort based on five imaging features: APHE, peritumoral enhancement, peritumoral hypointensity, T1rt-20min, and tumor margin. The nomograms and calibration curves are presented in **Figure 8**, which showed that the calibration curves for Ki-67 expression in the three cohorts all well-matched with standard lines. The C-index of the nomogram for Ki-67 expression prediction was 0.919 (95% CI, 0.858–0.970) in the training cohort, 0.925 (95% CI, 0.821–1.000) in the validation cohort, and 0.850 (95% CI, 0.736–0.952) in the external validation cohort (**Table 5**).

**Table 5:** Predictive performance of preoperative Gd-EOB-DTPA-enhanced MRI features in Ki-67 expression prediction.

Models	AUC,C-index	Sensitivity (%)	Specificity (%)	Accuracy (%)	PPV (%)	NPV (%)
Training (n = 73)	0.919 (95% CI: 0.858∼0.970)	74.3%	94.7%	84.9%	92.8%	80.0%
Validation (n = 31)	0.925 (95% CI: 0.821∼1.000)	93.3%	81.2%	87.1%	82.4%	92.9%
External validation (n = 46)	0.850 (95% CI: 0.736∼0.952)	80.0%	79.2%	79.5%	76.2%	82.6%

## Discussion

Gd-EOB-DTPA can be absorbed by the liver cells through the organic anion transporter polypeptide (OATP) pathway, and it provides structural information of lesions and carries functional information in the hepatobiliary phase, due to its dual extracellular and hepatobiliary properties (20). T1 mapping technology is a non-invasive quantitative analysis method of the tissue T1 value, which has good repeatability and strong operability. Gd-EOB-DTPA-enhanced MR combined T1 mapping is believed to provide abundant diagnostic information. In our study, we retrospectively collected data of 148 consecutive patients with HCC, and we investigated the correlation with T1 mapping, ADC quantification parameters, and Ki-67 expression. Our data showed that T1rt-Pre and T1rt-20min were strongly positively correlated with Ki-67 (r = 0.627, r = 0.607, *P* < 0.001); the ADC value was moderately negatively correlated with Ki-67(r = -0.401, *P* < 0.001), as seen in previous studies (20). However, the ADC values were less correlated with Ki-67 than T1 mapping; the possible explanation could be that the ADC value can be affected by technical parameters, such as limited image quality with poor signal-to-noise ratio and low spatial resolution, motion and air artifacts, and misregistration artifacts on the ADC map (21). T1 mapping reflects the fixed characteristics of the tissue, is not limited by the scan sequence parameters, and is positively correlated with the concentration of the gadolinium contrast agent in the tissue. Therefore, the T1 mapping value is more accurate, which has also been confirmed in the liver function and liver fibrosis assessment (13–15). The author believes that when the proliferation of tumor cells is more active, the tumor cells are arranged more closely per unit volume, which results in a larger T1rt-pre value; when the proliferation of tumor cells is more active, the proportion of normal liver cells contained in the tumor is lower, and the absorption of Gd-EOB-DTPA is also reduced, resulting in a larger value of T1rt-20min. There is a certain correlation between Ki-67 expression and histopathological differentiation (22, 23). Is the T1 mapping value affected by histological differentiation? Therefore, in our study, the correlation between the T1rt-pre, T1rt-20min, and Ki-67 expressions in the different histological differentiations was further obtained. No matter in the low differentiation group or in the high differentiation group, T1rt-Pre and T1rt-20min had statistical difference between high and low expression of Ki-67 (*P* < 0.05). It can be seen that T1 mapping is not affected by the degree of histopathological differentiation when predicting high and low expressions of Ki-67.

The expression of Ki-67 in cancer has been intensively studied, and most studies have shown that Ki-67 is associated with the metabolic, genetic, or clinical-pathological features of HCC (5, 19, 20). Aktas’s study showed that Ki-67 was one of the independent prognostic factors of recurrence on patients who underwent liver transplant for HCC (24). Cao et al. revealed that HCC with high Ki-67 expression was more aggressive, and its recurrence-free survival and postoperative overall survival were significantly lower than those of HCC with low Ki-67 expression (6). With the rise of multidisciplinary and multimodal comprehensive treatment of HCC, the high risk of recurrence in HCC patients requires adjuvant therapy and careful follow-up. Hence, a non-invasive preoperative method to predict the Ki-67 status is needed to guide individualized HCC treatment and postoperative surveillance in clinical practice. Many studies have attempted to determine preoperative predictors of HCC with poor prognosis using Ki-67 (6–8), microvascular invasion (MVI) (25, 26), cytokeratin 19 (27, 28), or microvascular density (20). Although the results of these relevant studies have confirmed the relationship between these indicators and prognosis, no consensus has been reached. In terms of the relative molecular mechanism, except for Ki-67 which can be a marker of cellular proliferative activity, few studies have described other mechanisms. Currently, it is more acceptable to use Ki-67 as an important biomarker to reflect tumor cell proliferation and to assess prognosis in patients with HCC and other malignant tumors. However, there is no unified standard for the high and low expression groups of Ki-67 yet. In some studies, 10% (29–31) was used as the cutoff value of Ki-67 expression, but in others, 14% (32) or 35% (7) or 50% (20) was used, and we used 25% (6, 17–19) as the cutoff value because we believe that a cutoff that is too low or too high will lead to selectivity bias for inclusion criteria due to the under- or overestimation of Ki-67 expression. At the same time, our data showed a difference in the overall early recurrence rate between the two groups (*P* < 0.001). The high Ki-67 expression group (>25%) showed a tendency to recur easily within 1 year after surgery.

In our study, MR qualitative characteristics such as APHE, tumor margin, tumor capsule, mosaic structure, arterial rim enhancement, peritumoral hypointensity, and peritumoral enhancement were statistically different between the high Ki-67 group and low Ki-67 group in the internal cohort (*P* < 0.05). However, among the above qualitative characteristics, there was no statistical difference in APHE and tumor capsule between the high Ki-67 expression group and low Ki-67 expression group in the external validation cohort. Among these meaningful MR characteristics in our study, peritumoral hypointensity, peritumoral enhancement, tumor capsule, and tumor margin mainly reflected the relatively dense cell structure and infiltrative growth types of the tumor to the surrounding structures. Peritumoral hypointensity and peritumoral enhancement probably relate to the known hypothesis of invasion of the surrounding structures especially the minute portal branch occlusion and the hemodynamic changes existing in compensatory arterial hyperperfusion and decreased portal flow, which may lead to altered expression of OATP or MRP2 receptors (33, 34). However, the statistically different MR characteristics developed in our study were not included in the regression model, such as mosaic structure, arterial rim enhancement, and tumor capsule. Mosaic structure could be related to the inhomogeneity of the tumor, while arterial rim enhancement pattern is rarely observed in HCCs and is more commonly observed in intrahepatic cholangiocarcinomas or metastases (27). It is noteworthy that APHE was statistically different between the two groups (*P* < 0.05) and included in the nomogram, while it was not a significant predictor of high Ki-67 expression. During hepatocarcinogenesis, changes in the tumor’s blood supply often lead to APHE. However, in some benign lesions, such as atypical hemangioma, it can also present with the typical enhancement pattern of APHE (35). This study did not conduct further analysis of the APHE subtypes, but Cunha’s study (36) showed that there are still differences in observers’ perceptions of APHE subtypes (37), and no unified understanding has been formed. Quantitative assessment can directly reflect histological features. In our study, T1rt-pre and T1rt-20min in the high Ki-67 group were higher than those in the low Ki-67 group (*P* < 0.001), while the ADC value in the high Ki-67 group was lower than that in the low Ki-67 group (*P* < 0.001). However, the ADC value was not included in the regression model.

Finally, the significant independent predictors of Ki-67 expression included peritumoral enhancement, peritumoral hypointensity, T1rt-20min, and tumor margin. Although APHE was included in the regression, it was not an independent predictor. For Ki-67 expression, a nomogram was established based on five imaging features: APHE, peritumoral enhancement, peritumoral hypointensity, T1rt-20min, and tumor margin in the training cohort. The combined nomogram yielded an incremental performance in predicting the Ki-67 expression of HCC; the C-index was 0.919 (95% CI, 0.858–0.970) in the training, 0.925 (95% CI, 0.821–1.000) in the validation cohort, and 0.850 (95% CI, 0.736–0.952) in the external validation group.

In summary, the author believes that gadoxetate disodium-enhanced MRI combined with T1 mapping quantitative technology can better evaluate the expression of Ki-67. However, there are several limitations in this study: (1) This is a retrospective study with a relatively small sample size, and there may be selection bias. (2) At present, there is currently no standardized Ki-67 expression level threshold in HCC and there are differences in various studies that we defined 25% as the cutoff value. Therefore, it is necessary to study the differences of different groups with a large sample of multiple centers and compare the diagnostic efficacy of different groups. (3) ROI is drawn manually, and there may be measurement errors. (4) Multiple liver cancers (>2) were excluded from this study. Because this study is a retrospective study, it is impossible to guarantee the one-to-one correspondence between the imaging images of multiple lesions and the pathological images. However, multiple liver cancers are not uncommon in clinical practice. A prospective study was conducted in conjunction with colleagues from hepatobiliary surgery and pathology departments.

## Conclusion

In conclusion, this study shows that the T1 relaxation time measured by Gd-EOB-DTPA-enhanced MRI T1 mapping has a strong positive correlation with Ki-67 expression in HCC, and our established nomogram has good predictive performance for a non-invasive preoperative prediction of Ki-67 expression in HCC.

## Data availability statement

The original contributions presented in the study are included in the article/supplementary material. Further inquiries can be directed to the corresponding authors.

## Ethics statement

This study was reviewed and approved by the ethics committee of each participating hospital. The patients/participants provided their written informed consent to participate in this study.

## Author contributions

Acquisiton of the data of the external validation group of our work: JF. All the other authors listed have made substantial, direct and intellectual contribution to the article and approved the submitted version.

## Funding

The study was supported by the grants of the science and technology planning project of Foshan (2020001005216) and Guangdong Medical Science and Technology Research Fund (A2021483).

## Acknowledgments

The authors thank the patients and their families for their participation in the study.

## Conflict of interest

The authors declare that the research was conducted in the absence of any commercial or financial relationships that could be construed as a potential conflict of interest.

## Publisher’s note

All claims expressed in this article are solely those of the authors and do not necessarily represent those of their affiliated organizations, or those of the publisher, the editors and the reviewers. Any product that may be evaluated in this article, or claim that may be made by its manufacturer, is not guaranteed or endorsed by the publisher.
